# Limited Effects of an *eIF2α* ^**S51A**^ Allele on Neurological Impairments in the 5xFAD Mouse Model of Alzheimer's Disease

**DOI:** 10.1155/2015/825157

**Published:** 2015-03-26

**Authors:** Katharina Paesler, Kan Xie, Moritz M. Hettich, Magdalena E. Siwek, Devon P. Ryan, Susanne Schröder, Anna Papazoglou, Karl Broich, Ralf Müller, Astrid Trog, Alexander Garthe, Gerd Kempermann, Marco Weiergräber, Dan Ehninger

**Affiliations:** ^1^German Center for Neurodegenerative Diseases (DZNE), Ludwig Erhard Allee 2, 53175 Bonn, Germany; ^2^Federal Institute for Drugs and Medical Devices (BfArM), Kurt Georg Kiesinger Allee 3, 53175 Bonn, Germany; ^3^Department of Psychiatry and Psychotherapy, University of Cologne, Kerpener Straße 62, 50937 Köln, Germany; ^4^Institute of Molecular Psychiatry, University of Bonn, Sigmund Freud Straße 25, 53125 Bonn, Germany; ^5^German Center for Neurodegenerative Diseases (DZNE), Fetscherstraße 105, 01307 Dresden, Germany

## Abstract

Alzheimer's disease (AD) has been associated with increased phosphorylation of the translation initiation factor 2*α* (eIF2*α*) at serine 51. Increased phosphorylation of eIF2*α* alters translational control and may thereby have adverse effects on synaptic plasticity, learning, and memory. To analyze if increased levels of p-eIF2*α* indeed promote AD-related neurocognitive impairments, we crossed 5xFAD transgenic mice with an *eIF*2*α* ^*S*51A^ knock-in line that expresses the nonphosphorylatable eIF2*α* variant *eIF*2*α* ^*S*51A^. Behavioral assessment of the resulting mice revealed motor and cognitive deficits in 5xFAD mice that were, with the possible exception of locomotor hyperactivity, not restored by the *eIF*2*α* ^*S*51A^ allele. Telemetric intracranial EEG recordings revealed no measurable effects of the *eIF*2*α* ^*S*51A^ allele on 5xFAD-associated epileptic activity. Microarray-based transcriptome analyses showed clear transcriptional alterations in 5xFAD hippocampus that were not corrected by the *eIF*2*α* ^*S*51A^ allele. In contrast to prior studies, our immunoblot analyses did not reveal increased levels of p-eIF2*α* in the hippocampus of 5xFAD mice, suggesting that elevated p-eIF2*α* levels are not a universal feature of AD models. Collectively, our data indicate that 5xFAD-related pathologies do not necessarily require hyperphosphorylation of eIF2*α* to emerge; they also show that heterozygosity for the nonphosphorylatable *eIF*2*α* ^*S*51A^ allele has limited effects on 5xFAD-related disease manifestations.

## 1. Introduction

Alzheimer's disease (AD) is associated with progressive cognitive and neurological impairments. Synaptic dysfunction downstream of toxic amyloid species is thought to play a major role in altered brain function and cognitive impairments in AD [1].

A growing body of literature suggests that translational regulatory mechanisms are disrupted in AD [2–4]. More specifically, AD has been associated with elevated phosphorylation levels of the eukaryotic translation initiation factor 2*α* (eIF2*α*). Hyperphosphorylation of eIF2*α* has been observed in postmortem samples derived from subjects affected by AD [5–7]. Studies in AD mouse models that are based on rare familial mutations associated with high risk for AD (i.e., mice overexpressing mutant amyloid precursor protein and/or presenilin) have indicated that eIF2*α* hyperphosphorylation can be seen in some of these disease models as well [6–8]. eIF2*α* is phosphorylated in the context of cellular stress responses, such as the endoplasmic reticulum (ER) unfolded protein response (UPR); increased eIF2*α* phosphorylation then leads to a general inhibition of protein synthesis [9]. In addition, elevated eIF2*α* phosphorylation favors the translational expression of certain mRNAs, including activating transcription factor 4 (ATF4) [10].

*De novo* protein synthesis is well known to play import roles in the establishment of long-lasting synaptic plasticity and the formation of long-term memory [11, 12]. Hyperphosphorylation of eIF2*α* may interfere with protein synthesis-dependent forms of plasticity and memory formation by inhibiting* de novo* protein synthesis [13]. Additionally, eIF2*α* hyperphosphorylation may perturb synaptic plasticity and memory formation by suppressing cAMP response element binding protein- (CREB-) dependent gene expression via upregulation of ATF4 [13]. These considerations suggest that p-eIF2*α*-mediated translational and transcriptional effects may contribute to altered plasticity and cognitive dysfunction in AD. Indeed, it has been reported that the genetic removal of either of two different eIF2*α* kinases (PRKR-like endoplasmic reticulum kinase, Perk, and general control nonderepressible 2, Gcn2) restores plasticity and memory impairments in an APP/PS1 mouse model of AD [14].

We, here, set out to explore the role of eIF2*α* phosphorylation in Alzheimer's pathogenesis by crossing the 5xFAD mouse model [15] with an *eIF*2*α* ^S51A^ knock-in line [16], in which eIF2*α* cannot be phosphorylated on the mutant allele due to substitution of serine at residue 51 by alanine. While most mouse model studies have looked at kinases upstream of eIF2*α* [14, 17], we were interested in analyzing the effects of a nonphosphorylatable *eIF*2*α* ^S51A^ allele on disease progression in an AD mouse model. eIF2*α* phosphorylation globally inhibits protein synthesis and thereby attenuates the flow of new protein into the endoplasmic reticulum under conditions of ER stress [16]. Mouse embryonic fibroblasts (MEFs) derived from *eIF*2*α* ^S51A/S51A^ animals lack a global repression of protein synthesis, as well as an induction of UPR-inducible genes during ER stress [16]. As a consequence, viability under ER stress is much reduced in *eIF*2*α* ^S51A/S51A^ cells compared to wild-type control cells [16]. Homozygous *eIF*2*α* ^S51A^ mutant mice were found to die after birth, likely due to extended hypoglycemia caused by defective gluconeogenesis [16]; heterozygous *eIF*2*α* ^S51A^ mutants do not exhibit compromised viability. 5xFAD mice overexpress (under control of Thy1 promoter) both mutant human* APP* with the Swedish (K670N, M671L), Florida (I716V), and London (V717I) familial Alzheimer's disease (FAD) mutations and human* PSEN1* harboring two FAD mutations (M146L and L286V) [15]. These animals show early and progressive amyloid deposits (beginning at 2 months), gliotic and inflammatory changes, neuronal loss, and neurocognitive impairments [15].

## 2. Material and Methods

### 2.1. Animals

5xFAD mice (genetic background: B6/SJL) overexpress mutant forms of human* APP* (the Swedish mutation: K670N, M671L; the Florida mutation: I716V; the London mutation: V717I) and mutant* PSEN1* (M146L, L286V) [15]. *eIF*2*α* ^+/S51A^ mice (genetic background: C57BL/6J) were generated as previously described [16]. 5xFAD mice were crossed with *eIF*2*α* ^+/S51A^ mice, yielding animals of four different genotypes: wild-type, 5xFAD, 5xFAD;*eIF*2*α* ^+/S51A^, and *eIF*2*α* ^+/S51A^. Our studies were performed using heterozygous *eIF*2*α* ^S51A^ mutant mice because homozygous *eIF*2*α* ^S51A^ mutants die shortly after birth [16] and are therefore not suitable to address the aims of the present paper.

One cohort of animals was generated for behavioral and neurological assessments. Tests were conducted in the following order/at the following age of the animals: open field (8 months, 11 months), wire hang test (10-11 months), tail suspension test (10-11 months), Morris water maze (12 months), and contextual fear conditioning (13-14 months). Ages of animals used for electrophysiological, biochemical, and gene expression analyses are provided in the respective sections below (see below). The present study was approved by “Landesamt für Natur, Umwelt und Verbraucherschutz Nordrhein-Westfalen” (Recklinghausen, Germany).

### 2.2. Tail Suspension Test

Assessment of pathological hindlimb clasping was performed using the tail suspension test. Hindlimb movements in the test were assigned to one of the four following categories: 1 = normal hindlimb movements, 2 = intermittent clasping of one hindlimb, 3 = intermittent clasping of both hindlimbs, and 4 = enduring clasping of both hindlimbs. Statistical analysis was performed by ordered logistic regression with the factors of 5xFAD genotype (5xFAD transgenes present versus absent) and eIF2*α* genotype (*eIF*2*α* ^+/S51A^ versus* eIF2α* ^+/+^).

### 2.3. Wire Hang Test

Mice were placed on a wire cage lid, which was turned upside down and positioned about 25 cm above an empty cage. Latency to fall was recorded with a maximum duration of 10 min. Mice received one trial per day over a period of 3 days (latencies were averaged across these trials). Statistical analysis was performed using two-way ANOVA with the between-subjects factors 5xFAD genotype (5xFAD transgenes present versus absent) and eIF2*α* genotype (*eIF*2*α* ^+/S51A^ versus* eIF2α* ^+/+^).

### 2.4. Open Field

To assess motor activity in a novel environment, mice were placed in an open field (27.5 cm × 27.5 cm × 20 cm) for 20 min on each of 3 consecutive days. The distance travelled was recorded using the EthoVision XT video tracking system (Noldus, Wageningen, Netherlands) and an average across all 3 sessions was calculated for each animal. Statistical analysis was performed using two-way ANOVA with the between-subjects factors 5xFAD genotype (5xFAD transgenes present versus absent) and eIF2*α* genotype (*eIF*2*α* ^+/S51A^ versus* eIF2α* ^+/+^).

### 2.5. Morris Water Maze

Mice were trained on a hidden version of the Morris water maze (Ø 135 cm). In this task animals learned to find an escape platform (Ø 10 cm) hidden underneath the water surface in a constant location of the pool. Mice received 6 training trials per day for 7 consecutive days. Training trials were completed when the animal had reached the escape platform or when 60 s were elapsed, whichever came first. During training trials, animals were started from randomly alternating starting positions. If animals did not manage to get on the escape platform within 60 s, they were gently guided to the escape platform. There was a 15 s posttrial period on the escape platform. To test how well the animals had learned the position of the escape platform, we gave a probe trial at the end of training day 7. During the probe trial the platform was removed from the pool and the swim pattern was analyzed (with respect to quadrant occupancy and target crossings). After completion of the 7 days of hidden training, we gave one day of cued training (6 trials), during which the platform position was indicated by a visible cue. Swim patterns of mice were recorded using the EthoVision XT video tracking system (Noldus, Wageningen, Netherlands). Time spent in quadrants and target crossings were analyzed using the built-in features of the software. Further analyses were done based on the raw time-tagged xy-coordinates using Matlab (The Mathworks). Search strategies were classified according to parameters described in a previous study [18], originally based on [19]. Search strategies were defined by no more than two quantitative parameters that were chosen to reflect the unique abstract properties of a given strategy and that were not dependent on the specific pool dimensions used. Statistical analysis of the groups was done by three-way repeated-measures ANOVA with the between-subjects factors 5xFAD genotype (5xFAD transgenes present versus absent) and eIF2*α* genotype (*eIF*2*α* ^+/S51A^ versus* eIF2α* ^+/+^) and one of the following within-subjects factors: training trials (for the analysis of the escape latency curves); quadrants (for the analysis of probe trial data, i.e., target crossings and quadrant occupancy, resp.).

### 2.6. Contextual Fear Conditioning

Mice were placed in a conditioning chamber (Med Associates, St. Albans, Vermont, USA) for 3 min and received foot shocks (0.75 mA, 2 s) after minutes 1 and 2. One day later, for testing, mice were again placed in the conditioning chamber for a period of 3 min. Freezing behavior and activity levels during the baseline (i.e., first minute on the training day) and the test were recorded and analysed using Video Fear Conditioning software (Med Associates, St. Albans, Vermont, USA). Due to differences in baseline activity levels between groups (data not shown), we do not report freezing scores but report activity suppression ratios instead, which were calculated as follows: activity_test_/(activity_baseline_ + activity_test_). Statistical comparison of the groups was performed by two-way ANOVA with the between-subjects factors 5xFAD genotype (5xFAD transgenes present versus absent) and eIF2*α* genotype (*eIF*2*α* ^+/S51A^ versus* eIF2α* ^+/+^).

### 2.7. Radiotelemetric EEG Recordings

Mice (>10 months of age) were anesthetized by intraperitoneal injection containing ketamine (KetanestR, Parke-Davis/Pfizer, Germany)/xylazine (RompunR 2%, Bayer Vital, Germany) at 100/10 mg/kg. For measuring of the electroencephalogram (EEG), TL11M2-F20-EET 2-channel transmitters (technical specification: 3.9 g, 1.9 cc; Data Science International (DSI), USA) were implanted into a subcutaneous pouch on the back of the animals. The first channel was used to target the primary motor cortex region (M1). A differential epidural electrode was placed at the following stereotaxic coordinates: (M1-) lead bregma +1 mm, lateral of bregma 1.5 mm (left hemisphere). For deep brain recordings targeting the hippocampal CA1 region, the differential electrode of channel 2 was positioned as follows: (CA1-) lead, bregma −2 mm, lateral of bregma 1.5 mm (right hemisphere), dorsoventral (depth) 1.5 mm. For both channels, epidural reference electrodes were placed at bregma −6 mm, lateral of bregma 1 mm (left hemisphere), and bregma −6 mm, lateral of bregma 1 mm (right hemisphere). Electrodes were fixed at the neurocranium using glass ionomer cement (Kent Express, UK), and the scalp was closed using over and over sutures (Ethilon, 6-0). A detailed description of the implantation procedure is available in a previous publication [20]. For postoperative pain management carprofen (5 mg/kg; Rimadyl, Pfizer, Germany) was administered subcutaneously to the animals.

Following a 10-day recovery period, simultaneous video-EEG recordings from the motor cortex (M1) and the hippocampal CA1 region were performed for 48 h using Dataquest ART 4.2 software (DSI) at a sampling rate of 500 Hz with no* a priori* filter cut-off.

For further analyses, data were processed using Neuroscore 2.1 (DSI). Seizure analyses and calculations were performed using Neuroscore Spike Train Detector, that is, a seizure detection module. Seizure protocols contained total number of episodes and spikes, spike frequency, total spike train duration, and shortest and longest spike train duration.

### 2.8. Histology and Immunostaining

Mice (13 months old) were anaesthetized by intraperitoneal injection of ketamine (100 mg/kg) and xylazine (7 mg/kg). For perfusion, the bloodstream was rinsed with sterile 0.9% sodium chloride and organs were fixed using 4% paraformaldehyde (PFA) in phosphate buffered saline (PBS). Brains were dissected and postfixed in 4% PFA in PBS overnight at 4°C and dehydrated in 30% sucrose in PBS at 4°C. Coronal brain sections of 40 *μ*m thickness were cut using a sliding microtome (Leica, Wetzlar, Germany) and were stored in cryoprotectant buffer (0.05 M phosphate buffer, 25% glycerol, and 25% ethylene glycol) at −20°C. Brain sections spaced 240 *μ*m apart were transferred into Tris-buffered saline (TBS), washed twice for 5 min and incubated in 0.6% H_2_O_2_ in TBS for 30 min. Sections were then washed three times in TBS for 5 min. For blocking, sections were incubated for 30 min in TBS-plus (TBS, 0.1% Triton X-100, and 3% donkey serum). Next, sections were incubated with primary antibody (rabbit anti-choline acetyltransferase (ChAT), 1 : 100, Millipore, Darmstadt, Germany) in TBS-plus for 48 h at 4°C, washed twice in TBS for 5 min, and blocked in TBS-plus for 45 min. This was followed by a 1-hour incubation step (at room temperature) in TBS-plus containing biotin-conjugated secondary antibody (donkey anti-rabbit, 1 : 500, Jackson ImmunoResearch Laboratories, West Grove, PA, USA). Sections were then washed three times in TBS for 5 min and incubated in 3,3′-diaminobenzidine (DAB) for 2 min using the DAB Substrate Kit (Roche, Mannheim, Germany). To stop the peroxidase reaction, sections were washed in tap water and TBS. Next, sections were mounted on glass slides, dehydrated in an ascending series of alcohol (twice 70%, once 96%, and twice 100% Ethanol), cleared in xylene, and covered with a coverslip. Stereological analysis of brain sections was performed using a bright field microscope Eclipse 90i (Nikon, Düsseldorf, Germany). Immunoreactive neurons within the medial septum (MS) and vertical limb of the diagonal band of Broca (VDB) were quantified using the software Stereo Investigator (MBF Bioscience, Magdeburg, Germany). Statistical analysis was performed via two-way ANOVA with the between-subjects factors 5xFAD genotype (5xFAD transgenes present versus absent) and eIF2*α* genotype (*eIF*2*α* ^+/S51A^ versus* eIF2α* ^+/+^).

### 2.9. Immunoblot Analysis

Hippocampal samples were taken from 14- to 15-month-old mice after cervical dislocation and snap-frozen in liquid nitrogen. Each sample was homogenized in 250 *μ*L lysis buffer containing RIPA buffer, Phospho-Stop (Roche, Mannheim, Germany), Protease-Inhibitor (Roche, Mannheim, Germany), 50 mM sodium fluoride, and 5 mM sodium orthovanadate and incubated on a rotator for 30 min at 4°C. Samples were centrifuged at 14000 rpm for 15 min at 4°C. Protein concentration was determined using the Pierce BCA protein assay kit (Thermo Fisher Scientific, Bonn, Germany). If not stated otherwise, 15 *μ*g protein was loaded on 10% tris glycine gels (APP, ChAT, eIF2*α*) or 16% tris tricine gels (CTF) for PAGE. Proteins were transferred onto a polyvinylidene difluoride (PVDF) membrane and incubated in TBS with 10% Western Blocking Reagent (Roche, Mannheim, Germany) for 1 h. Incubation of PVDF membrane in primary antibody (rabbit anti-eIF2*α*, 1 : 2000, Cell Signaling, Danvers, MA, USA; rabbit anti-p-eIF2*α*, 1 : 2000, Cell Signaling, Danvers, MA, USA; rabbit anti-APP, 1 : 1000, Cell Signaling, Danvers, MA, USA; rabbit anti-CTF, 1 : 1000, Sigma-Aldrich, Taufkirchen, Germany; goat anti-ChAT, 1 : 1000, Millipore, Darmstadt, Germany; mouse anti-A*β*, clone 6E10, 1 : 1000, Covance, Princeton, NJ, USA; mouse anti-*α*-tubulin, 1 : 20000, Abcam, Cambridge, UK; mouse anti-*β*-actin, 1 : 5000, MP Biomedicals, Solon, OH, USA) in TBS with 5% Western Blocking Reagent was carried out overnight at 4°C. After multiple washing steps in TBS with 0.1% Tween-20, membranes were incubated in horseradish peroxidase-conjugated secondary antibody (donkey anti-rabbit IgG, 1 : 1000, Jackson ImmunoResearch Laboratories, West Grove, PA, USA; donkey anti-mouse IgG, 1 : 1000, Jackson ImmunoResearch Laboratories, West Grove, PA, USA; donkey anti-goat IgG, 1 : 10000, Jackson ImmunoResearch Laboratories, West Grove, PA, USA) in TBS with 5% Western Blocking Reagent (for 1 h at room temperature or overnight at 4°C). Next, membranes were washed several times in TBS with 0.1% Tween-20. Immunosignals were detected using enhanced chemiluminescence (Amersham ECL Western Blotting Detection Reagents, GE Healthcare, Munich, Germany, or SuperSignal West Femto Maximum Sensitivity Substrate, Thermo Fisher Scientific, Bonn, Germany) and were quantified using a Chemidoc XRS imager (Bio-Rad, Munich, Germany). Densitometric analysis was performed using Image Lab software (Bio-Rad, Munich, Germany). Proteins were normalized to *α*-tubulin or *β*-actin, and phosphorylated proteins were normalized to their respective total proteins. eIF2*α* and p-eIF2*α* were detected on different blots together with the respective loading controls. Statistical analysis was accomplished by *t*-test or two-way ANOVA with the between-subjects factors 5xFAD genotype (5xFAD transgenes present versus absent) and eIF2*α* genotype (*eIF*2*α* ^+/S51A^ versus* eIF2α* ^+/+^), as appropriate.

### 2.10. A*β* Enzyme-Linked Immunosorbent Assays (ELISA)

Hippocampal homogenates in RIPA buffer (as described above) were used for quantitative analysis of the two abundant species of amyloid *β* (A*β*), A*β*40 and A*β*42, using ELISA kits (Life Technologies, Carlsbad, CA, USA) according to manufacturer's instructions. Amounts of A*β* peptides were subsequently normalized to protein concentration of the respective sample.

### 2.11. RNA Extraction and Affymetrix Microarray Procedures

Microarray experiments were carried out using GeneChip Mouse Exon 1.0 ST v1 Expression Chip (Affymetrix). These exon arrays contain 6.553.600 probes with a coverage density of 4 probes for each exon of all known and predicted genes of the mouse genome. Mouse hippocampal tissue of 14- to 15-month-old mice was processed with the RNeasy Micro Kit (Qiagen, Hilden, Germany) according to the manufacturer's instructions. RNA integrity was determined with Agilent's Bioanalyzer and only samples with integrity numbers between 9.2 and 10 were chosen for downstream applications. Arrays were washed and stained according to the manufacturer's recommendations. Labeled and purified cDNA was fragmented (5.5 *μ*g) and subsequently hybridized to the arrays before scanning in a GeneChip 3000 7G scanner (Affymetrix). Normalization to the median of all samples, background correction as well as statistical analysis, was performed with GeneSpringGX software (Agilent technologies). An implemented GC-RMA algorithm was applied on all chips to summarize probe level information. Microarray data were analyzed using moderated *t*-tests. Benjamini Hochberg FDR was employed and comparisons with a *P* value <0.02 were considered statistically significant. Differentially regulated transcripts with a fold change (FC) greater than 1.6 were then subjected to hierarchical clustering analysis in order to visualize gene expression changes across groups. Array data are available in the GEO database under GSE50521. DAVID [21, 22] was used to carry out gene ontology enrichment analyses in the gene set differentially expressed between 5xFAD and wild-type controls. In addition, we used ingenuity pathway analysis to help define common features of genes differentially expressed in 5xFAD hippocampus.

### 2.12. Cell Culture

Confluent SHSY5Y wild-type cells in 6-well plates were treated with vehicle (ddH_2_O) for 15 min or with 2 mM dithiothreitol (DTT) for 15 or 60 min. After treatment cells were washed with PBS, detached with Trypsin/EDTA, and centrifuged at 500 ×g for 5 min at 4°C. Pelleted cells were resuspended in 100 *μ*L lysis buffer (TBS, 1% Triton X-100, Protease-Inhibitor (Roche, Mannheim, Germany), and Phospho-Stop (Roche, Mannheim, Germany)) and incubated on ice for 30 min. Cell homogenates were centrifuged at 10000 ×g for 10 min at 4°C. The supernatant was used for immunoblot analysis.

## 3. Results

In order to test if increased eIF2*α* phosphorylation contributes to cognitive dysfunction and disease progression in 5xFAD mice, we crossed these mice with an *eIF*2*α* ^S51A^ knock-in line [16], in which eIF2*α* cannot be phosphorylated on one allele due to substitution of serine at residue 51 by alanine.

**Figure 1:**
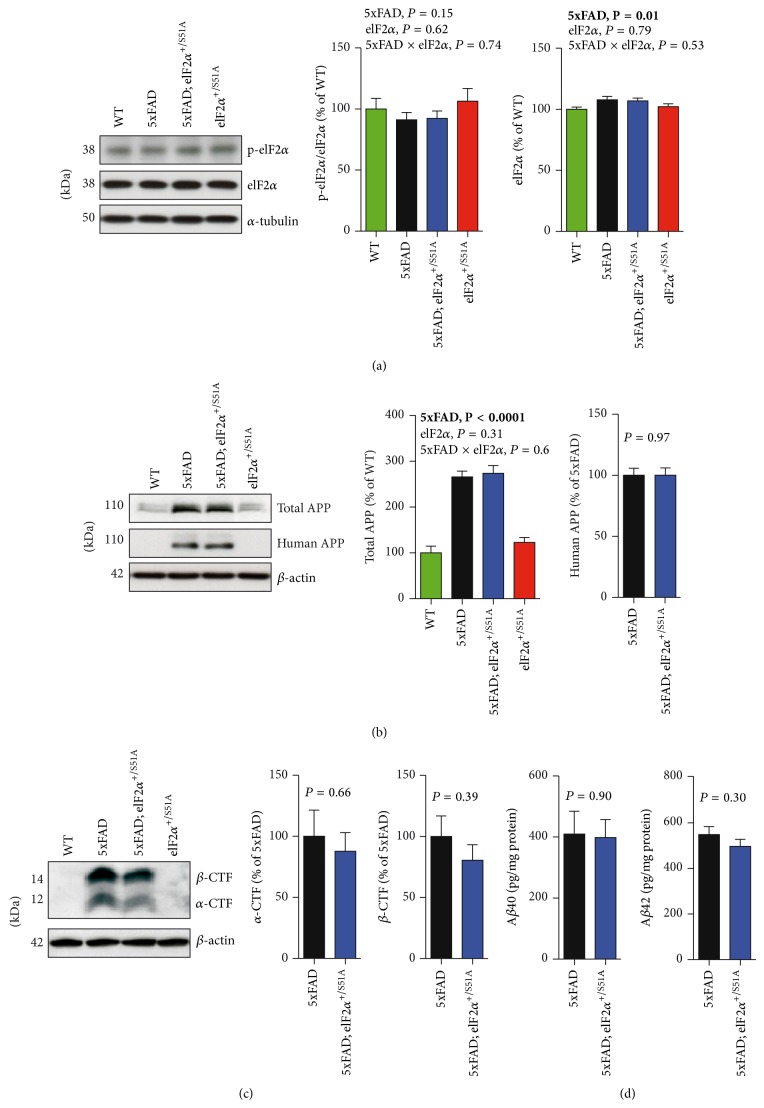
The *eIF*2*α* ^S51A^ mutation had no measurable effects on eIF2*α* phosphorylation and APP processing. Shown are representative immunoblots of (a) p-eIF2*α* and total eIF2*α*, (b) human APP and total APP, and (c) *α*-CTF and *β*-CTF all prepared from hippocampal homogenates, along with the respective quantitative densitometric data (WT, *n* = 6 mice; 5xFAD, *n* = 7 mice; 5xFAD;*eIF*2*α* ^S51A^, *n* = 6 mice; *eIF*2*α* ^S51A^, *n* = 5 mice). (d) Concentrations of abundant A*β*-species, A*β*40 and A*β*42, in 5xFAD hippocampal homogenates were determined using ELISA (5xFAD, *n* = 7 mice; 5xFAD;*eIF*2*α* ^S51A^, *n* = 6 mice). All data are presented as mean ± SEM. Data were analyzed using two-way ANOVAs with the between-subjects factors 5xFAD genotype/eIF2*α* genotype (effect of 5xFAD transgenes; effect of eIF2*α* genotype; 5xFAD × eIF2*α* interaction) and *t*-tests (5xFAD versus 5xFAD;*eIF*2*α* ^S51A^) as appropriate. Statistically significant differences (*P* < 0.05) are denoted by bold font.

We performed immunoblot analyses to measure the abundance of p-eIF2*α* (i.e., eIF2*α* phosphorylated at serine 51), total eIF2*α*, APP, and APP cleavage products in lysates prepared from hippocampal tissue of 5xFAD, 5xFAD;*eIF*2*α* ^+/S51A^, and *eIF*2*α* ^+/S51A^ animals as well as wild-type controls. Unexpectedly but in agreement with one other recent study [23], our analyses showed no significant effect of the 5xFAD transgenes on p-eIF2*α* levels (Figure 1(a); two-way ANOVA with between-subjects factors 5xFAD genotype and eIF2*α* genotype, effect of 5xFAD genotype, *P* = 0.15). Additional experiments showed the expected [24] increase in p-eIF2*α* abundance in cells treated with 2 mM DTT (see Supplementary Figure 1 in the Supplementary Material available online at http://dx.doi.org/10.1155/2015/825157; one-way ANOVA with treatment as between-subjects factor, *P* = 0.01), indicating that our experimental settings were suited, in principle, to detect eIF2*α* hyperphosphorylation. Consistent with previously published work [7], the 5xFAD transgenes affected total eIF2*α* levels with slightly increased eIF2*α* abundance in animals bearing the 5xFAD transgenes (Figure 1(a); two-way ANOVA with between-subjects factors 5xFAD genotype and eIF2*α* genotype, effect of 5xFAD genotype, *P* = 0.01). The eIF2*α* phosphorylation status did not differ significantly between heterozygous carriers of the *eIF*2*α* ^S51A^ mutation and animals carrying two wild-type* eIF2α* alleles (Figure 1(a); two-way ANOVA with between-subjects factors 5xFAD genotype and eIF2*α* genotype, effect of eIF2*α* genotype, *P* = 0.62). These biochemical findings suggest that an excessive eIF2*α* phosphorylation is not a universal feature of AD mouse models, such as 5xFAD mice, and they also indicate that the heterozygous *eIF*2*α* ^S51A^ mutation is not necessarily sufficient to suppress hippocampal eIF2*α* phosphorylation.

Further immunoblot analyses showed no clear effects of the *eIF*2*α* ^S51A^ mutation on the abundance of total APP (Figure 1(b); two-way ANOVA with between-subjects factors 5xFAD genotype and eIF2*α* genotype, effect of eIF2*α* genotype, *P* = 0.31), human APP (Figure 1(b); *t*-test, 5xFAD versus 5xFAD;*eIF*2*α* ^+/S51A^, *P* = 0.97), and APP cleavage products (Figure 1(c); *α*-CTF, *t*-test, 5xFAD versus 5xFAD;*eIF*2*α* ^+/S51A^, *P* = 0.66; *β*-CTF, *t*-test, 5xFAD versus 5xFAD; *eIF*2*α* ^+/S51A^, *P* = 0.39). ELISA analyses showed no group differences regarding hippocampal A*β*40 and A*β*42 concentrations (Figure 1(d); A*β*40, *t*-test, 5xFAD versus 5xFAD;*eIF*2*α* ^+/S51A^, *P* = 0.90; A*β*42, *t*-test, 5xFAD versus 5xFAD;*eIF*2*α* ^+/S51A^, *P* = 0.30).

In line with these biochemistry results, our behavioral, electrophysiological, and gene expression analyses revealed limited effects of the *eIF*2*α* ^S51A^ allele on disease phenotypes present in 5xFAD mice, as outlined below.

**Figure 2:**
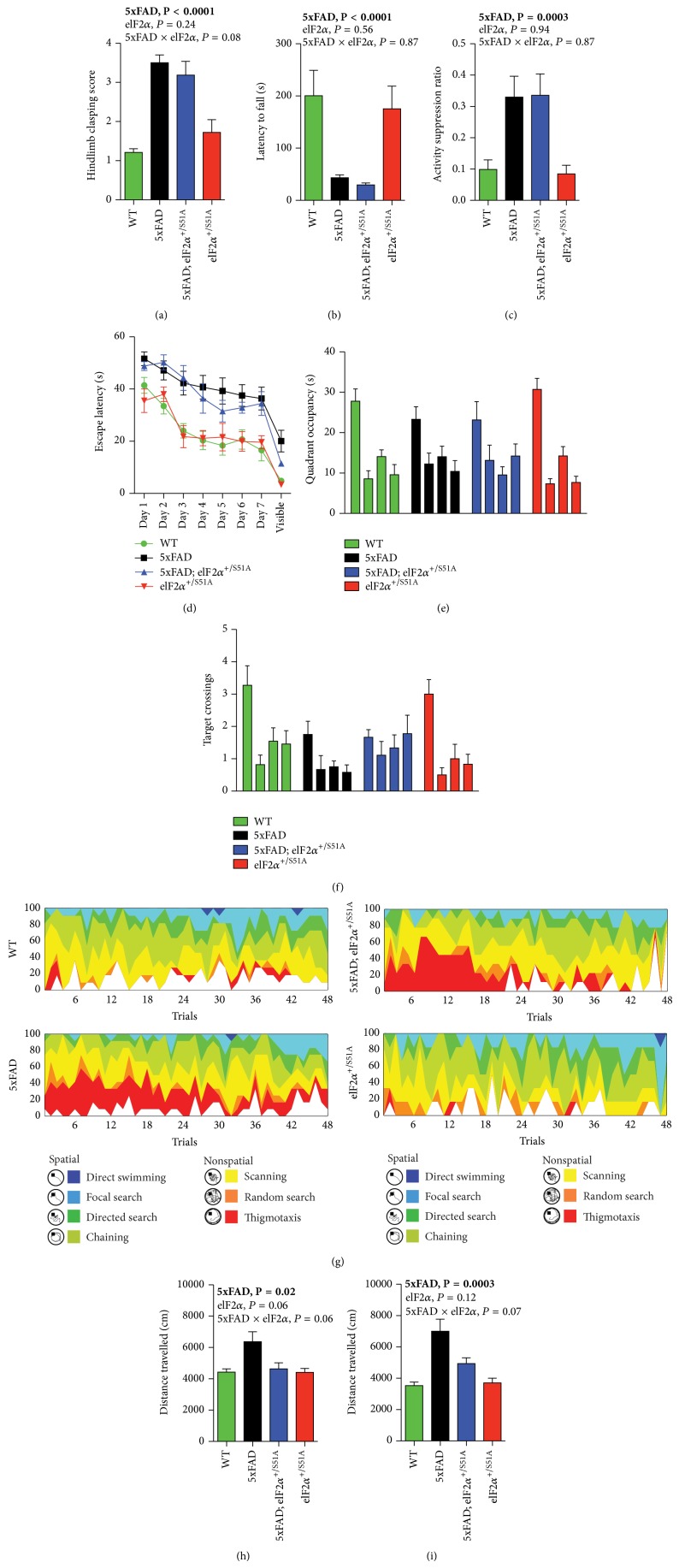
The *eIF*2*α* ^S51A^ allele had limited effects on most neurological phenotypes but restored hyperactivity in 5xFAD mice. (a) Hindlimb clasping scores, as assessed in the tail suspension test (WT, *n* = 11 mice; 5xFAD, *n* = 12 mice; 5xFAD;*eIF*2*α* ^S51A^, *n* = 9 mice; *eIF*2*α* ^S51A^, *n* = 6 mice). (b) Latencies to fall in the context of a wire hang test (WT, *n* = 11 mice; 5xFAD, *n* = 13 mice; 5xFAD;*eIF*2*α* ^S51A^, *n* = 11 mice; *eIF*2*α* ^S51A^, *n* = 8 mice). (c) Activity suppression ratios in a context fear conditioning paradigm (WT, *n* = 11 mice; 5xFAD, *n* = 12 mice; 5xFAD;*eIF*2*α* ^S51A^, *n* = 9 mice; *eIF*2*α* ^S51A^, *n* = 6 mice). ((d)–(g)) Results of an assessment of spatial learning and memory in the Morris water maze (WT, *n* = 11 mice; 5xFAD, *n* = 12 mice; 5xFAD;*eIF*2*α* ^S51A^, *n* = 9 mice; *eIF*2*α* ^S51A^, *n* = 6 mice). (d) Escape latencies during training trials. (e) Quadrant occupancy and (f) target crossings measures obtained during a probe trial given after the completion of training day 7. For each genotype, bars represent (from left to right) target quadrant, opposite quadrant, adjacent right quadrant, and adjacent left quadrant. (g) Results of an extended swim path analysis: for each group, the proportion of animals in the respective search categories is plotted against training trial. ((h),(i)) Distance travelled in two open field experiments performed at either 8 months ((h); WT, *n* = 11 mice; 5xFAD, *n* = 13 mice; 5xFAD; *eIF*2*α* ^S51A^, *n* = 11 mice; *eIF*2*α* ^S51A^, *n* = 8 mice) or 11 months of age ((i); WT, *n* = 11 mice; 5xFAD, *n* = 13 mice; 5xFAD;*eIF*2*α* ^S51A^, *n* = 11 mice; *eIF*2*α* ^S51A^, *n* = 8 mice), respectively. Data were analyzed using two-way ANOVAs with the between-subjects factors 5xFAD genotype and eIF2*α* genotype ((a)–(c), (h), (i)) or using three-way ANOVAs with the between-subjects factors 5xFAD genotype and eIF2*α* genotype and the within-subjects factor trial (d) or quadrant ((e), (f)). Statistically significant differences (*P* < 0.05) are denoted by bold font. For additional information regarding the results of statistical analyses, see main text. Bar and line graphs show means ± SEM.

In brief, the *eIF*2*α* ^S51A^ allele did not appear to ameliorate pathological hindlimb clasping in 5xFAD mice (Figure 2(a); ordered logistic regression, effect of 5xFAD transgenes, *P* < 0.0001; effect of eIF2*α* genotype *P* = 0.24; 5xFAD genotype × eIF2*α* genotype interaction, *P* = 0.081), nor was there any apparent effect on motor impairments as measured in the wire hang test (Figure 2(b); two-way ANOVA with between-subjects factors 5xFAD genotype and eIF2*α* genotype, effect of 5xFAD genotype, *P* < 0.0001; effect of eIF2*α* genotype, *P* = 0.56; 5xFAD × eIF2*α* interaction, *P* = 0.87). We analyzed learning and memory using a context fear conditioning paradigm (Figure 2(c)) and the Morris water maze (Figures 2(d)–2(g)). In context fear conditioning, 5xFAD mice showed higher activity suppression scores upon testing, indicative of associative learning impairments in these animals (Figure 2(c); two-way ANOVA with between-subjects factors 5xFAD genotype and eIF2*α* genotype, effect of 5xFAD genotype, *P* = 0.0003), that were not influenced by the *eIF*2*α* ^S51A^ allele in any obvious way (Figure 2(c); two-way ANOVA with between-subjects factors 5xFAD genotype and eIF2*α* genotype, effect of eIF2*α* genotype, *P* = 0.94; 5xFAD × eIF2*α* interaction, *P* = 0.87).

In the Morris water maze, 5xFAD animals showed substantially higher escape latencies during training trials than controls (Figure 2(d); three-way repeated-measures ANOVA with 5xFAD genotype and eIF2*α* genotype as between-subjects factors and training trial as within-subjects factor, effect of 5xFAD transgenes, *P* < 0.0001; effect of eIF2*α* genotype, *P* = 0.76; 5xFAD × eIF2*α* interaction, *P* = 0.67), as well as a reduced number of target crossings during the probe trial given after completion of training (Figure 2(f); three-way repeated-measures ANOVA with 5xFAD genotype and eIF2*α* genotype as between-subjects factors and quadrant as within-subjects factor, 5xFAD genotype × quadrant interaction, *P* = 0.03; eIF2*α* genotype × quadrant interaction, *P* = 0.89; eIF2*α* genotype × 5xFAD genotype × quadrant interaction, *P* = 0.58). An extended strategy analysis, in the context of which behaviors during the training trials were classified into increasingly hippocampus-dependent (directed search, focal search, and direct swimming), as well as primarily hippocampus-independent (chaining, scanning, random search, and thigmotaxis) search categories, revealed an excessive use of less hippocampus-dependent strategies in animals with the 5xFAD transgenes (Figure 2(g)). The *eIF*2*α* ^S51A^ allele had no obvious modulatory effect on any of these 5xFAD water maze phenotypes (Figures 2(d)–2(g)).

Our behavioral analyses did, however, reveal one neurobehavioral 5xFAD phenotype that appeared to be restored by the *eIF*2*α* ^S51A^ allele (Figures 2(h) and 2(i)). 5xFAD animals showed pronounced motor hyperactivity in an open field assay (two-way ANOVA with 5xFAD genotype and eIF2*α* genotype as between-subjects factors, effect of 5xFAD genotype, *P* = 0.02 and 0.0003, resp.). 5xFAD-related hyperactivity appeared to be reduced in animals carrying the *eIF*2*α* ^S51A^ allele (two-way ANOVA with 5xFAD genotype and eIF2*α* genotype as between-subjects factors, 5xFAD ×* eIF2α* genotype, *P* = 0.06 for panel (h) and *P* = 0.07 for panel (i)).

The neurobiology underlying this possible *eIF*2*α* ^S51A^-mediated rescue of 5xFAD-related hyperactivity remains to be elucidated. Here, we considered one possibility which is that the *eIF*2*α* ^S51A^ allele modifies the degeneration of the cholinergic system in 5xFAD animals, which may contribute to hyperactive behaviors in AD mouse models [25–30]. Initial stereological cell counting showed neither a significant effect of the 5xFAD transgenes (two-way ANOVA with 5xFAD genotype and eIF2*α* genotype as between-subjects factors, effect of 5xFAD genotype, *P* = 0.74) nor an effect of the *eIF*2*α* ^S51A^ allele (two-way ANOVA with 5xFAD genotype and eIF2*α* genotype as between-subjects factors, effect of eIF2*α* genotype, *P* = 0.32) on the number of ChAT-positive neurons in the basal forebrain (Supplementary Figure 2). Immunoblot analyses of ChAT abundance in the hippocampus, one of the target areas that basal forebrain cholinergic neurons project into, however, revealed lower ChAT levels in animals carrying the 5xFAD transgenes (Supplementary Figure 2; two-way ANOVA with 5xFAD genotype and eIF2*α* genotype as between-subjects factors, effect of 5xFAD genotype, *P* = 0.002). The *eIF*2*α* ^S51A^ allele had no apparent effect on hippocampal ChAT protein abundance (two-way ANOVA with 5xFAD genotype and eIF2*α* genotype as between-subjects factors, effect of eIF2*α* genotype, *P* = 0.49) and also showed no significant interaction with the 5xFAD genotype (two-way ANOVA with 5xFAD genotype and eIF2*α* genotype as between-subjects factors, 5xFAD × eIF2*α* genotype interaction, *P* = 0.78), indicating that the *eIF*2*α* ^S51A^ allele did not protect against the hippocampal ChAT loss in 5xFAD mice.

**Figure 3:**
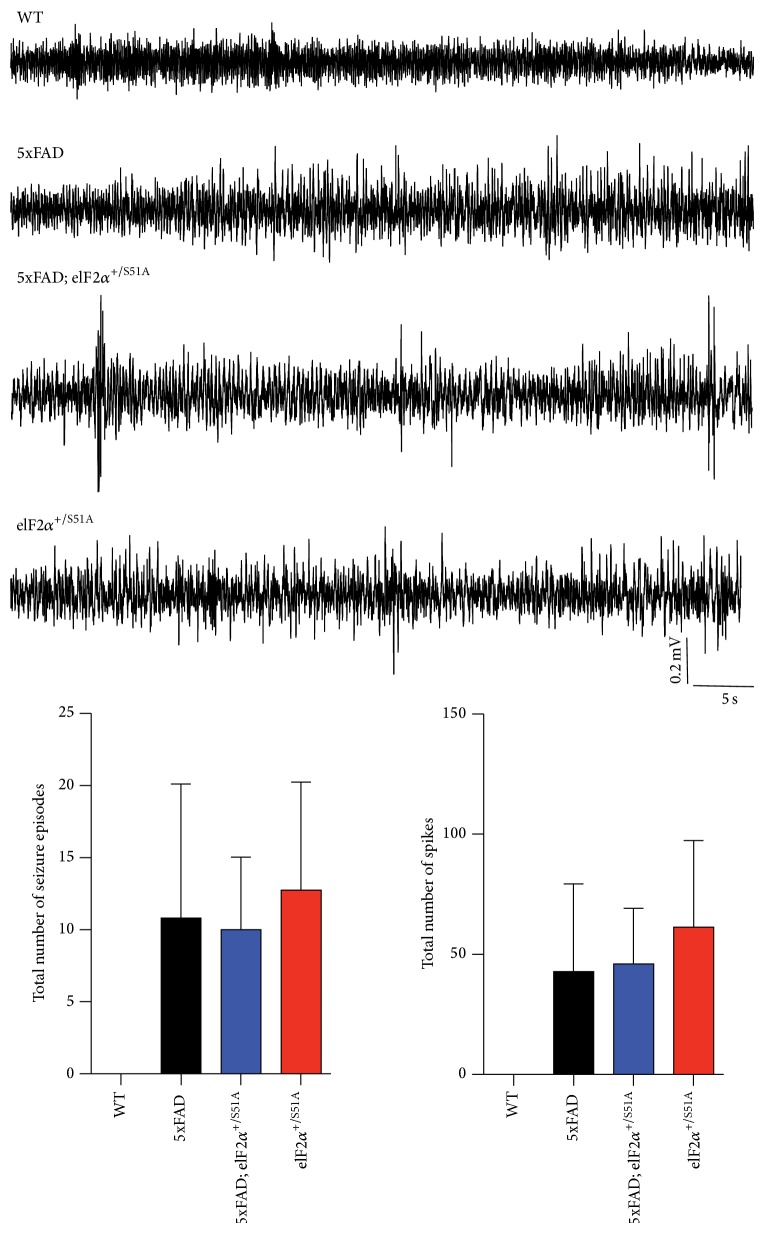
EEG recordings revealed nonconvulsive seizure activity in the motor cortex of animals carrying the 5xFAD transgenes and/or the *eIF*2*α* ^+/S51A^ allele. Radiotelemetric recordings from the primary motor cortex (M1) of wild-type, 5xFAD, 5xFAD;*eIF*2*α* ^+/S51A^, and *eIF*2*α* ^+/S51A^ mice (WT, *n* = 4 mice; 5xFAD, *n* = 6 mice; 5xFAD;*eIF*2*α* ^S51A^, *n* = 3 mice; *eIF*2*α* ^S51A^, *n* = 4 mice). Wild-type mice did not exhibit seizure activity, whereas all other genotypes showed episodes of spike, polyspike, and spike-wave activity. Shown are example traces, as well as a quantification of the number of seizure episodes and the number of spikes, respectively. Bar graphs show mean ± SEM.

Network hyperexcitability and seizures are important features of animal models of AD [31, 32]. We performed electrocorticographic (M1) and deep intrahippocampal CA1 EEG recordings in 5xFAD animals crossed into the *eIF*2*α* ^S51A^ background (Figure 3). Qualitative and quantitative seizure analysis using Neuroscore Seizure Module (DSI) revealed that animals of all genotypes with the exception of wild-type animals exhibited seizure activity in the M1 recording, although ictal discharges were not seen in the deep, intrahippocampal CA1 recording (in none of the groups; not shown). Video analysis revealed that none of the cortical seizures was associated with motoric exacerbation. Thus, we observed predominantly nonconvulsive seizure activity in 5xFAD mice. *eIF*2*α* ^+/S51A^ mice showed nonconvulsive seizure activity as well and introduction of the *eIF*2*α* ^S51A^ mutation in 5xFAD mice did not appear to modify the epileptic phenotype in any apparent way.

AD is associated with considerable transcriptional alterations in key brain areas [33–36]. Microarray analyses performed on hippocampal tissue of 5xFAD animals crossed into the *eIF*2*α* ^S51A^ background revealed a number of 5xFAD-related transcriptional alterations: statistical analysis revealed 106 genes differentially regulated between 5xFAD mice and wild-type controls (Supplementary Figure 3). Gene ontology analysis showed a substantial enrichment of immune-response related genes in this gene set (Supplementary Tables 1 and 2), which is in agreement with considerable inflammatory and immunological alterations observed in the context of cerebral amyloidosis. A comparison between 5xFAD and 5xFAD;*eIF*2*α* ^+/S51A^ animals revealed no differentially expressed genes, indicating that the *eIF*2*α* ^S51A^ mutation had no measurable effects on hippocampal transcriptional changes induced by the 5xFAD transgenes.

## 4. Discussion

In this study, we assessed the effects of a nonphosphorylatable* eIF2α* allele (*eIF*2*α* ^S51A^) on disease progression in the 5xFAD mouse model of familial AD. While profound pathology was evident in 5xFAD mice, these abnormalities remained (mostly) unmodified by the heterozygous *eIF*2*α* ^S51A^ mutation. This was the case for a wide range of molecular (APP expression and processing; gene expression), electrophysiological (EEG), and neurobehavioral (motor impairments; learning and memory impairments) features of the model.

Currently, contradictory reports exist regarding the eIF2*α* phosphorylation status in 5xFAD mice. Elevated p-eIF2*α* levels in the brain of 5xFAD mice have been published previously [7, 17], but more recent studies found no elevated phosphorylation of eIF2*α* in 5xFAD mice [23], which is in agreement with our observation of unaltered p-eIF2*α* levels in the hippocampus of 5xFAD mice. We currently do not know the factors that might account for these discrepant findings. Possibilities that need to be formally addressed in future studies include differences in genetic background, on which the 5xFAD mutations were kept, differences in the brain areas examined, and age at assessment. The 5xFAD breeders used to generate the animals of the present study were obtained on a mixed B6/SJL genetic background (this is expected to result in background composition varying from animal to animal) and were crossed to *eIF*2*α* ^+/S51A^ mice on a C57BL/6J background. Other studies employed 5xFAD animals on a mixed B6/SJL genetic background (again, with background composition varying from animal to animal) [7] or on a C57BL/6 background [17]. Age may be another important variable in modifying the effect that the 5xFAD genotype has on eIF2*α* hyperphosphorylation. Younger (i.e., 3–6 months old) 5xFAD animals showed only modest or no measurable hyperphosphorylation of eIF2*α* or related kinases (Perk) despite the presence of notable plaque pathology at this age [7, 37], indicating that pronounced eIF2*α* hyperphosphorylation, if at all present, may be a feature of more advanced stages of cerebral amyloidosis. Finally, when analyzing eIF2*α* phosphorylation status, it is particularly important to normalize p-eIF2*α* abundance to total eIF2*α* protein abundance because the 5xFAD genotype may be associated with an increased abundance of total eIF2*α* ([7] and Figure 1 of the present study).

While we did not observe measurable *eIF*2*α* ^+/S51A^-associated decrements in p-eIF2*α* abundance, others have reported moderate reductions in p-eIF2*α* abundance in *eIF*2*α* ^+/S51A^ mice [13, 23]. One possibility, again, is that this discrepancy is due to modulatory effects of genetic backgrounds that may have differed between these studies (C57BL/6J in [13]; nonstandardized, mixed B6/SJL backgrounds in the present study, as well as in [23]).

Ma et al. reported that conditional homozygous deletion of either of two different eIF2*α* kinases (Perk, Gcn2) in forebrain neurons improved spatial memory impairments in the APPswe/PSEN1dE9 mouse model of AD [14]. Deletion of either Perk or Gcn2 led to reduced p-eIF2*α* levels in this model [14], suggesting that these genetic manipulations were more effective in suppressing eIF2*α* phosphorylation than the heterozygous *eIF*2*α* ^S51A^ mutation used in the present study. Accordingly, limited effects on neurological impairments observed in our study could be related to a less effective suppression of p-eIF2*α* levels by the heterozygous *eIF*2*α* ^S51A^ mutation. Homozygous *eIF*2*α* ^S51A^ mutants could not be examined because of early postnatal lethality associated with this genotype [16]. In addition, it is possible that we would have been able to detect more pronounced rescue effects of the *eIF*2*α* ^S51A^ mutation on earlier stages of the disease process (i.e., in younger animals) in the model employed. Moreover, amyloid pathology progresses at a faster pace in 5xFAD animals (used in the present study) than in the model examined by Ma et al. (APPswe/PSEN1dE9 mice) and, hence, neurological impairments in the 5xFAD model may generally be less accessible to amelioration than those in APPswe/PSEN1dE9 mice.

One exception to the notion that the *eIF*2*α* ^+/S51A^ genotype did not influence 5xFAD phenotypes was the observation of restored motor hyperactivity in 5xFAD;*eIF*2*α* ^+/S51A^ mice. Elevated motor activity levels are a consistent feature of AD rodent models [38–41] and may also be observed in human individuals affected by the disorder [42]. Given the profound degeneration of basal forebrain cholinergic neurons in AD [43–46] and the role that basal forebrain cholinergic neurons play in the regulation of motor activity levels (ablation of basal forebrain cholinergic nuclei and anticholinergic pharmacological interventions increase motor activity in rats [30]), we asked whether the *eIF*2*α* ^S51A^ allele might restore hyperactivity by rescuing basal forebrain cholinergic neuron loss in 5xFAD mice. Our stereological analyses showed, however, no significant reduction of ChAT-immunoreactive neurons in the MS and VDB of 5xFAD mice, indicating that frank loss of cholinergic neurons was limited in the model at the age assessed. Nevertheless, immunoblot analyses were sensitive enough to pick up clear 5xFAD-related reductions in ChAT protein levels in one of the target areas of basal forebrain cholinergic nuclei, namely, the hippocampus, which is consistent with prior studies in transgenic AD mouse models [25–29]. 5xFAD and 5xFAD;*eIF*2*α* ^+/S51A^ mice did not differ significantly in their hippocampal ChAT protein levels, indicating that the *eIF*2*α* ^S51A^ allele did not rescue the aberrant cholinergic system of 5xFAD mice. Besides cholinergic dysfunction, AD is associated with perturbations in other neurotransmitter systems, such as the serotonergic system [47–53], and serotonergic alterations may potentially contribute to AD-associated locomotor hyperactivity [51, 54–56]. Future studies should further assess the possible mechanistic underpinnings of the *eIF*2*α* ^S51A^ effect on 5xFAD-related motor hyperactivity and assessments of the serotonergic system may provide one possible starting point for such studies.

Our finding that the *eIF*2*α* ^+/S51A^ allele had no effects on the APP abundance in 5xFAD mice is in line with a recent report that used a combination of genetic approaches and found no effect of p-eIF2*α* reductions on A*β*-dependent APP, as well as BACE1 levels [23], indicating that A*β*-dependent increases in APP and BACE1 levels do not involve eIF2*α*-dependent translational mechanisms.

In conclusion, our study revealed few effects of the *eIF*2*α* ^+/S51A^ genotype on disease progression in the 5xFAD mouse model of AD with the exception of a possible amelioration of 5xFAD-related motor hyperactivity. Our data indicate that *eIF*2*α* ^S51A^ heterozygosity is not necessarily sufficient to measurably suppress eIF2*α* phosphorylation in the 5xFAD mouse model employed. Future studies need to further elaborate on the specific conditions, under which a 5xFAD genotype may be associated with eIF2*α* hyperphosphorylation.

## Supplementary Material

Supplementary Figure 1. Enhanced phosphorylation of eIF2α in SHSY5Y cells incubated with DTT. Shown are p-eIF2α and eIF2α immunoblots performed on lysates of SHSY5Y cells that had been treated with vehicle or 2mM DTT for 15 or 60 min (n = 2 samples per condition). Densitometric quantification revealed the expected increase in eIF2α phosphorylation in SHSY5Y cells incubated with DTT. P-values refer to results of posthoc Tukey tests.
Supplementary Figure 2. The cholinergic system was not involved in the eIF2α+/S51A-related restoration of hyperactivity in 5xFAD mice. (A) Shown are micrographs of ChAT-immunoreactive neurons in the basal forebrain of wild-type, 5xFAD, 5xFAD;eIF2α+/S51A and eIF2α+/S51A mice. Scale bar = 150 μm. Stereological quantification of ChAT-immunoreactive neurons in the MS and VDB of the basal forebrain demonstrated no significant differences between the four groups of mice (n = 3 mice per group). (B) Shown are immunoblots of ChAT from hippocampal homogenates of wild-type, 5xFAD, 5xFAD;eIF2α+/S51A and eIF2α+/S51A mice (n = 5-7 mice per group). Data were analyzed using two-way ANOVAs with the between-subjects factors 5xFAD genotype and eIF2α genotype. Statistically significant differences (p < 0.05) are denoted by bold font. Bar graphs show mean ± SEM.
Supplementary Figure 3. The eIF2αS51A allele had limited effects on transcriptional dysregulation in 5xFAD mice. Differentially regulated transcripts (Benjamini Hochberg FDR, p < 0.02) with a fold change higher than 1.6 were subjected to hierarchical clustering to visualize gene expression changes between groups (n = 3 mice per genotype).
Supplementary Table 1. A gene ontology analysis (DAVID) revealed a significant enrichment of immune-related transcripts in gene set differentially expressed in 5xFAD hippocampus.
Supplementary Table 2. Ingenuity pathway analysis also indicated a substantial enrichment of genes related to immune and inflammatory processes in the gene set differentially expressed in 5xFAD hippocampus.
